# Molecular Epidemiology of *Cryptosporidium* spp., *Giardia duodenalis*, and *Enterocytozoon bieneusi* in Guizhou Angus Calves: Dominance of Angus Cattle-Adapted Genotypes and Zoonotic Potential of *E. bieneusi*

**DOI:** 10.3390/microorganisms13081735

**Published:** 2025-07-25

**Authors:** Peixi Qin, Zhuolin Tao, Kaizhi Shi, Jiaxian Zhao, Bingyan Huang, Hui Liu, Chunqun Wang, Jigang Yin, Guan Zhu, Simone M. Cacciò, Min Hu

**Affiliations:** 1 National Key Laboratory of Agricultural Microbiology, College of Veterinary Medicine, Huazhong Agricultural University, Wuhan 430070, China; peixiqin@webmail.hzau.edu.cn (P.Q.); taozhuolin@webmail.hzau.edu.cn (Z.T.); shkzjjp@163.com (K.S.); jiaxian06280826@163.com (J.Z.); huangbingyan@webmail.hzau.edu.cn (B.H.); liuhui45@webmail.hzau.edu.cn (H.L.); wangchunqun@mail.hzau.edu.cn (C.W.); 2 State Key Laboratory for Diagnosis and Treatment of Severe Zoonotic Infectious Diseases, Key Laboratory for Zoonosis Research of the Ministry of Education, Institute of Zoonosis, College of Veterinary Medicine, Jilin University, Changchun 130012, China; yinjg@jlu.edu.cn (J.Y.); zhuguan@jlu.edu.cn (G.Z.); 3 Department of Infectious Diseases, Istituto Superiore di Sanità, 00161 Rome, Italy; simone.caccio@iss.it

**Keywords:** zoonotic parasites, *Cryptosporidium* spp., *Giardia duodenalis*, *Enterocytozoon bieneusi*, molecular epidemiology

## Abstract

Limited molecular data exist on zoonotic parasites *Cryptosporidium* spp., *Giardia duodenalis*, and *Enterocytozoon bieneusi* in Angus calves from Guizhou, China. This study constitutes the first molecular epidemiological survey of these pathogens in this region. 817 fecal samples from Angus calves across 7 intensive beef farms (Bijie City). Nested PCR methods targeting SSU rRNA (*Cryptosporidium* spp.), *gp60* (*Cryptosporidium bovis* subtyping), *bg*/*gdh*/*tpi* (*G. duodenalis*), and ITS (*E. bieneusi*) coupled with DNA sequencing were employed. DNA sequences were analyzed against the NCBI. database. Statistical differences were assessed via a generalized linear mixed-effects model. *Cryptosporidium* spp. prevalence 23.5% (192/817; 95% CI 28.1–34.6%), with *C. bovis* predominating 89.6% (172/192; 95% CI 84.4–93.5%) and six subtypes (XXVIa-XXVIf). Highest infection in 4–8-week-olds 29.9% (143/479; 95% CI 25.8–34.1%) (*p* < 0.01). *G. duodenalis*: 31.3% (256/817; 95% CI 28.1–34.6%) positive, overwhelmingly assemblage E 97.6% (6/256; 95% CI 0.9–5.0%), zoonotic assemblage A was marginal 0.7% (6/817; 95% CI 0.3–1.6%). Farm-level variation exceeded 10-fold (e.g., Gantang: 55.0% (55/100; 95% CI 44.7–65.0%) vs. Tieshi: 4.9% (5/102; 95% CI 1.6–11.1%). *E. bieneusi*: prevalence 19.7% (161/817; 95% CI 17.0–22.6%), exclusively zoonotic genotypes BEB4: 49.7% (80/161; 95% CI 41.7–57.7%); I: 40.4% (65/161; 95% CI 32.7–48.4%). Strong diarrhea association (*p* < 0.01) and site-specific patterns (e.g., Guanyindong: 39.2%). While *Giardia* exhibited the highest prevalence (31.3%) with minimal zoonotic risk, *Enterocytozoon*—despite lower prevalence (19.7%)—posed the greatest public health threat due to exclusive circulation of human-pathogenic genotypes (BEB4/I) and significant diarrhea association, highlighting divergent control priorities for these enteric parasites in Guizhou calves. Management/Public health impact: Dominant zoonotic *E. bieneusi* genotypes (BEB4/I) necessitate: 1. Targeted treatment of 4–8-week-old Angus calves. 2. Manure biofermentation (≥55 °C, 3 days), and 3. UV-disinfection (≥1 mJ/cm^2^) for karst water to disrupt transmission in this high-humidity region.

## 1. Introduction

*Cryptosporidium* spp. (phylum Apicomplexa), *Giardia duodenalis* (phylum Metamonada), and *Enterocytozoon bieneusi* (phylum Microsporidia) are all capable of infecting both humans and a variety of animal hosts [1,2,3]. *Cryptosporidium* spp. is primarily transmitted via contaminated water and food and can cause diarrhea in children under 2 years of age and calves under 1 month of age, potentially leading to death in severe cases. In immunocompetent hosts, it often manifests as asymptomatic or self-limited diarrhea, while in immunocompromised patients, it can cause severe diarrhea [4]. Globally, taxonomic studies have identified 44 *Cryptosporidium* species, and *C. parvum*, *C. andersoni*, *C. ryanae*, and *C. bovis* represent the epidemiologically predominant species in cattle [5,6]. *Giardia duodenalis*, transmitted via the same contaminated water and food as *Cryptosporidium* spp., affects 280 million people annually, causing diarrhea, malabsorption, and weight loss [7]. It has 8 assemblages, labeled A through H, with zoonotic *G. duodenalis* assemblages A and B being commonly detected in cattle [8]. *Enterocytozoon bieneusi* is a microsporidian parasite that is divided into 11 genetic groups (1–11) based on its ribosomal RNA (ITS region). Group 1 (Zoonotic): Contains genotypes (A, BEB15) found in many animals (livestock, pets, wildlife) and the environment. This group is the main source of human infections, showing significant animal-to-human (zoonotic) transmission. Group 2 (Human-Adapted): Includes genotypes (notably BEB4, I, J) that mostly infect humans, especially those with weak immune systems. While sometimes found in animals, they are strongly adapted to humans or spread between humans (anthroponotic). Groups 3–11 (Host-Specific): Primarily infect specific animals with limited known spread to humans. Understanding the classification and zoonotic potential of *E. bieneusi* is crucial for public health and epidemiological studies [9].

Globally, the epidemiology of *Cryptosporidium* spp., *G. duodenalis,* and *E. bieneusi* in cattle has been extensively documented across continents such as Asia, Europe, North America, South America, and Oceania [10,11,12]. In China, these pathogens have been studied in multiple provinces, including Henan, Shaanxi, Beijing, Shanghai, Hubei, Hebei, Tianjin, Qinghai, Sichuan, Guangdong, Jiangsu, Tibet Autonomous Region, Gansu, Shandong, Yunnan, Anhui, Inner Mongolia, Xinjiang, Shanxi, Heilongjiang, and Ningxia [13,14,15,16,17,18,19,20,21,22,23,24,25,26,27,28,29,30,31,32]. However, there is a noticeable absence of data from Guizhou Province, China. Bijie City of Guizhou Province serves as a major hub for Angus cattle farming, hosting numerous large-scale cattle ranches that constitute a significant center for livestock production in Southwestern China. This study aims to address this gap by determining the prevalence and genotypic distribution of *Cryptosporidium* spp., *G. duodenalis,* and *E. bieneusi* in the fecal samples of Angus calves in Bijie City of Guizhou Province, thereby contributing to the national epidemiological dataset on *Cryptosporidium* spp., *G. duodenalis,* and *E. bieneusi* in China.

## 2. Materials and Methods

### 2.1. Sample Collection

In our study, a total of 817 Angus calves’ fecal samples were collected from Bijie City, Guizhou Province (26°21′~27°46′ N and 103°36′~106°43′ E), a closed fattening farm. Management: Corn-soybean diet (6:4 ratio), ad libitum feeding; biannual FMD/Influenza A vaccination; quarterly ivermectin deworming; semi-open cement-floor housing (4 m^2^/head). Key equipment: Hydraulic restraint stand and electronic scale (±0.5 kg accuracy).

**Figure 1:**
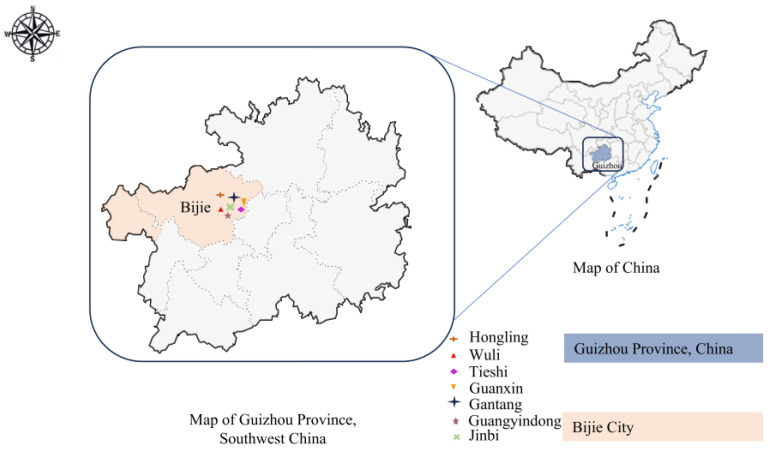
Geographic distribution of Angus cattle sampling sites in Guizhou Province, China.

In August 2023, we collected 79 and 75 fecal samples at Wuli and Hongling farms, respectively. In March 2024, we obtained 663 fecal samples from Angus calves across seven farms in the city, including Wuli (89), Hongling (99), Tieshi (102), Guanxin (93), Gantang (100), Guangyindong (97), and Jinbi (83) (Figure 1). Importantly, the samples collected in 2024 at these Wuli and Hongling farms were from cohorts, differing from the animals studied in 2023. Constrained by resources and funding, summer sampling was conceived as a preliminary investigation for seasonal comparison. Wuli and Hongling were guided by established collaborative partnerships. This study prioritized analyzing the impact of spring breeding protocols on prevalence rates, therefore concentrating resources on comprehensive spring data acquisition. Minimum sample sizes were calculated based on historical *Cryptosporidium* spp. 22.7% (35/154; 95% CI 16.4–30.2%), *G. duodenalis* 20.1% (31/154; 95% CI 14.1–27.3%), and *E. bieneusi* 12.3% (19/154; 95% CI 7.6–18.6%), with prevalence rates at 5% margin of error, yielding 270, 247, and 166 samples, respectively. The study’s 663 collected samples substantially exceeded these requirements. The study population consisted of 817 Angus calves aged 0–3 months, comprising 178 male and 639 female Angus calves, as well as 107 0–2-week age, 200 2–4-week age, 479 4–8-week age, 31 8–12-week age, 93 diarrhea, and 724 no-diarrhea fecal samples. Fecal samples were collected from Angus calves under 3 months of age using aseptic rectal swabbing techniques, with one sample per animal. Inclusion criteria comprised: No anthelmintic treatment within 30 days; absence of acute disease (core temperature ≤ 39.5 °C); no vaccination within 14 days prior; non-orphaned status. Through computer-generated randomization, 10% of eligible calves were selected for sampling. All sampled subjects met the predefined criteria irrespective of baseline health status. Detailed sampling data are presented in Table S1. Following collection, all samples underwent immediate storage at 4 °C. Subsequent transport to the laboratory utilized SF Express cold chain logistics, after which samples were maintained at 4 °C in refrigerated storage until DNA extraction. Each farm can accommodate up to 10,000 Angus cattle and is divided into three functional zones: the production area, the lactation area, and the fattening area. In the production area, each shed houses one Angus calf, while the other zones practice mixed grazing. Cows are free to move within any area, promoting natural behavior and reducing stress.

### 2.2. DNA Extraction and PCR Analysis

DNA extraction was performed using the TIANamp DNA stool Kit (TIANGEN, Beijing, China) following the manufacturer’s instructions. Briefly, 0.2 g of fecal material were homogenized using 1 mm grinding beads in the FastPrep^®^-24 Instrument (MP Biomedicals, Santa Ana, CA, USA) for 60 s at a frequency of 5.5 m/s, and this process was repeated. The extracted DNA was then eluted with 80 μL of molecular-grade water and stored at −20 °C for future analysis. In this study, *Cryptosporidium* spp. was detected using a nested PCR method targeting the 830 bp SSU rRNA gene. The PCR amplification was performed using the DNA polymerase and 2 × SanTaq PCR Master Mix (Sangon Biotech, Shanghai, China), following the reagent and condition protocols established in a previous study [33] and subtyping of *C. bovis* test as mentioned in the article [34]. *G. duodenalis* was identified and assemblaged based on sequence analysis of the glutamate dehydrogenase (*gdh*) gene, β-galactosidase (*bg*) gene, and triosephosphate isomerase (*tpi*) gene, respectively [35]. Detection of *E. bieneusi* used internal transcribed spacer (ITS), and the method from the prior study [36]. Negative controls, consisting of molecular-grade water, were included in each PCR analysis to ensure accuracy. The secondary PCR products were examined by electrophoresis on a 1.5% agarose gel and visualized following ethidium bromide staining. The primers and annealing temperatures employed in this study are listed in Table 1

### 2.3. Sequence Analysis

Positive PCR amplicons were sequenced by Qingke Biology (Beijing, China). The DNA sequences obtained in this study were checked using TBtools-II v. 2.125, then blasted against reference sequences from GenBank, and finally analyzed using the Neighbor-Joining analysed by MEGA v.11.0.13 software.

### 2.4. Statistical Analysis

All statistical analyses were performed using SPSS v. 27.0.1 software. The generalized linear mixed-effects model was used to evaluate the statistical differences in *Cryptosporidium* spp., *G. duodenalis,* and *E. bieneusi* infections among pre-weaned Angus calves, stratified by location, season, age, diarrhea, and gender. Statistical significance was determined with 95% confidence intervals. The *p* < 0.05 indicates statistical significance.

## 3. Results

### 3.1. Cryptosporidium bovis Dominance: 89.6% Prevalence with Geographic Clustering

In this study, of the 817 fecal samples collected from Bijie City, Guizhou Province, 192 tested positive for *Cryptosporidium* spp., via nested PCR targeting the SSU rRNA gene. The overall prevalence of *Cryptosporidium* species infection was 23.5% (192/817; 95% CI 28.1–34.6%), with the following distribution among the *Cryptosporidium* species: *C. andersoni* 2.1% (4/192; 95% CI 0.6–5.2%), *C. bovis* 89.6% (172/192; 95% CI 84.4–93.5%), and *C. ryanae* 7.8% (15/192; 95% CI 4.4–12.6%) (Table 2, Figure S1). Geographical variations in *Cryptosporidium* species prevalence were particularly notable across the 7 sampled farms. The highest infection rate was observed in Tieshi, 33.3% (34/102; 95% CI 24.3–43.3%), while Hongling showed the lowest prevalence, 12.1% (21/174; 95% CI 7.7–17.9%). Intermediate prevalence rates were observed in Wuli, 27.9% (47/168; 95% CI 21.3–35.3%), Guanxin (18/93; 95% CI 11.9–28.9%), Gantang, 26.0% (26/100; 95% CI 17.7–35.7%), Guanyindong, 28.9% (28/97; 95% CI 20.1–39.0%), and Jinbi, 21.7% (18/83; 95% CI 13.4–32.1%) (Table 2). Statistical analysis revealed significant differences in *Cryptosporidium* spp. positive rates across the 7 sites (*p* < 0.001). One of the Angus cattle from Hongling was found to be co-infected with *C. bovis* and *C. ryanae*. This study reveals significant differences in the dominant subtypes of *C. bovis* across various farms. The sample positive rate was 23.4% (155/663; 95% CI 20.2–26.8%) in spring and 24.0% (37/154; 95% CI 17.5–31.6%) in summer, with no significant difference observed between the seasons (*p* = 0.963 > 0.05). Furthermore, no significant interaction effect was found between location and season (*p* = 0.531 > 0.05), indicating their independent influences (Table 2).

Statistical analysis revealed significant variations in *Cryptosporidium* species prevalence across different age groups of Angus calves (*p* < 0.01). The highest positive rate was detected in 4–8-week-old Angus calves, 29.9% (143/479; 95% CI 25.8–34.1%), followed by Angus calves aged 2–4 weeks, with 21.5% (43/200; 95% CI 16.0–27.8%). Positive rates decreased to12.9% (4/31; 95% CI 3.6–29.8%) for Angus calves aged 8–12 weeks and were lowest, 4.7% (2/107; 95% CI 1.5–10.6%) in those less than 2 weeks old. Of the total samples, 22.6% (21/93; 95% CI 14.6–32.4%) of diarrhea cases tested positive, compared to 23.6% (171/724; 95% CI 20.6–26.9%) of non-diarrhea cases. There was no significant difference in the positive rates between diarrhea samples and non-diarrhea samples (*p* = 0.817 > 0.05). Males exhibited a lower positive rate, 17.9% (32/178; 95% CI 12.6–24.4%), compared to females, 25.0% (160/639; 95% CI 21.7–28.6%), with this difference being statistically significant (*p* = 0.032 < 0.05) (Table 2).

In this study, 6 subtypes of *C. bovis* were identified. On Hongling, Tieshi, and Guanxin farms, the subtypes XXVIf and XXVIe were commonly identified. On Hongling Farm, XXVIf was the predominant subtype. On Wuli Farm, four subtypes, XXVIb, XXVIc, XXVId, and XXVIe, were identified with relatively equal distribution, and a unique case of dual infection with XXVIb and XXVIc was detected. Guanyindong and Gantang farms share three *C. bovis* subtypes, XXVIa, XXVId, and XXVIf, but Guanyindong also had an additional subtype, XXVIe, which is not found in Gantang. XXVIa and XXVId were the most common subtypes in Guanyindong. On Jinbi Farm, the subtypes XXVIb and XXVIc were found, with XXVIc being the dominant subtype (Table 3, Figure S2).

### 3.2. Giardia duodenalis Assemblage E Prevails: Low Zoonotic Risk but High Farm-Level Variability

The overall prevalence of *G. duodenalis* infection across all study sites reached 31.3% (256/817; 95% CI 28.1–34.6%). Marked geographical variations were observed: Hongling recorded 18.9% (33/174; 95% CI 13.4–25.6%), Wuli 26.2% (44/168; 95% CI 19.7–33.5%), Tieshi 4.9% (5/102; 95% CI 1.6–11.1%), Guanxin 39.8% (37/93; 95% CI 29.8–50.5%), Gantan 55.0% (55/100; 95% CI 44.7–65.0%), Guanyindong 51.5% (50/97; 95% CI 41.2–61.8%), and Jinbi 38.6% (32/83; 95% CI 28.1–49.9%) (Table 4, Figure S1, Figure S2 and Figure S3). These regional differences demonstrated statistical significance (*p* < 0.001). The sample positive rate was 33.8% (224/663; 95% CI 30.2–37.5%) in spring and 20.8% (32/154; 95% CI 14.7–28.0%) in summer, with no significant difference observed between the seasons (*p* = 0.569 > 0.05). Furthermore, no significant interaction effect was found between location and season (*p* = 0.505 > 0.05), indicating their independent influences (Table 4). While zoonotic type A of *G. duodenalis* was detected in samples from Hongling, Wuli, and Guanxin farms, the infection rate was strikingly low, representing only 0.7% (6/817; 95% CI 0.3–1.6%) of total samples, among all the positive samples, the proportion of type A was 2.3% (6/256; 95% CI 0.9–5.0%). In contrast, type E was more prevalent in the Angus cattle population, 97.6% (250/256; 95% CI 95–99.1%). (Table 4). Infection rates varied significantly among different age Angus calves, with a statistically significant difference (*p* < 0.001). The highest infection rate, 37.2% (178/479; 95% CI 32.8–41.7%), was in 4–8-week-old Angus calves. Subsequent rates declined to 32.3% (10/31; 95% CI 16.7–51.4%) for 8–12 weeks, and 24.5% (49/200; 95% CI 18.7–31.1%) for 2–4 weeks. The lowest infection rate, 17.7% (19/107; 95% CI 11.0–26.3%), was found in Angus calves under 2 weeks. Diarrheal samples had a positive rate of 24.7% (23/93; 95% CI 16.3–34.8.3%), whereas non-diarrheal samples showed a higher positive rate of 32.2% (33/724; 95% CI 28.8–35.7%). However, the difference in positive rates between the diarrheal and non-diarrheal samples was not statistically significant (*p* = 0.166 > 0.05). Males exhibited a lower positive rate, 29.8% (53/178; 95% CI 23.2–37.1%), compared to females, 31.8% (203/639; 95% CI 28.2–35.5%), with this difference being no statistically significant (*p* = 0.629 > 0.05) (Table 4).

### 3.3. Enterocytozoon bieneusi Zoonotypes: BEB4/I Co-Circulation and Diarrhea Association

The overall prevalence of *E. bieneusi* was 19.7% (161/817; 95% CI 17.0–22.6%) (Table 5, Figure S6), with significant inter-location variation (*p* < 0.001). Notably, no positive cases were detected in Hongling and Guanxin farms. In contrast, positive cases were detected in other farms with positive rates ranging from 18.5% to 39.2%, showing the highest rate at 39.2% (38/97; 95% CI 29.4–49.6%) in Guanyindong and Gantang at 39.0% (39/100; 95% CI 29.4–49.3%), followed by Jinbi at 34.9% (29/83; 95% CI 24.8–46.2%), Tieshi at 23.5% (24/102; 95% CI 15.7–32.9%), and Wuli at 18.5% (31/168; 95% CI 12.9–25.2%). ITS sequencing analysis revealed four genotypes, including BEB4 49.7% (80/161; 95% CI 41.7–57.7%; 80/161), CHPM1 1.9% (3/161; 95% CI 0.4–5.3%), J 8.1% (13/161; 95% CI 4.4–13.4%), and I 40.4% (65/161; 95% CI 32.7–48.4%). The first three genotypes belong to Group 2 and are of the animal-adapted type, meaning they primarily infect animals but have also been detected in humans. The last genotype belongs to Group 1 and is a zoonotic genotype, which can be transmitted from animals to humans. Genotype J was only found in Wuli, and genotype CHPM1 was identified exclusively in Gantang. No new genotypes were detected. The sample positive rate was 21.4% (142/663; 95% CI 18.4–24.7%) in spring and 12.0% (19/154; 95% CI 7.6–18.6%) in summer, with no significant difference observed between the seasons (*p* = 0.843 > 0.05). Furthermore, no significant interaction effect was found between location and season (*p* = 0.843 > 0.05), indicating their independent influences (Table 5). The infection rates among Angus calves varied significantly by age (*p* < 0.001); the rates were as follows: the positivity rate increased progressively from 6.5% (7/107; 95% CI 2.7–13.0%) at 0–2 weeks to a peak of 26.1% (125/479; 95% CI 22.2–30.3%;) at 4–8 weeks of age; the positive rates were similar at 2–4 weeks, 12.5% (25/200; 95% CI 8.3–17.9%), and at 8–12 weeks, 12.9% (4/31; 95% CI 3.6–29.8%). The positive rate for diarrheal samples was 35.5% (33/93; 95% CI 25.8–46.1%), while for non-diarrheal samples, it was 17.7% (128/724; 95% CI 15.0–20.7%). Our study found a significant association between *E. bieneusi* infection and diarrhea in pre-weaned Angus calves (*p* < 0.001). Males exhibited a lower positive rate, 12.4% (22/178; 95% CI 7.9–18.1%), compared to females, 21.8% (139/639; 95% CI 18.6–25.2%), with this difference not being statistically significant (*p* = 0.065 > 0.05) (Table 5).

## 4. Discussion

### 4.1. Why Guizhou’s Infection Rates Surpass Sichuan and Yunnan: A Climate Lens?

The infection rate of *Cryptosporidium* species observed in our study was 23.5%, which aligns closely with the 22.5% prevalence reported in calves under 12 months of age across China from 2008 to 2018 [37]. This similarity extends to the prevalence of *C. bovis*, *C. ryanae*, and *C. andersoni* in pre-weaned calves [38]. However, infection rates can vary due to differing regional factor. In China, the regions with the highest infection rates are Taiwan, Inner Mongolia, Shandong, Hunan, and Qinghai; the regions with the lowest infection rates are Shanxi, Guangxi, Sichuan, Ningxia, and Gansu [39]. For example, in the southwestern region, infection rates in pre-weaned calves were 14.4% in Sichuan and 14.7% in Yunnan [20,40]. Regarding *G. duodenalis*, an infection rate of 31.3% was recorded, with the zoonotic assemblage A present at 0.7%. In contrast, the average *G. duodenalis* infection rate in Chinese cattle was reported to be 8.0% in 2022, with significant variation across provinces. For instance, in North China, the infection rate in Beijing was 1.7% [15]; in Northwest China, rates in Gansu, Ningxia, and Qinghai were 1.96% [24], 3.5% [41], and 8.1% [42], respectively; in South China, the rate in Guangdong was 74.2% [21]; in Central China, the rate in Hubei was 22.7% [17]; and in Southwest China, rates in Yunnan and Sichuan were 10.5% and 13.4%, respectively [20,26]. In pre-weaned Angus calves from Guizhou, an *E. bieneusi* infection rate of 19.7% was found, comparable to the global infection rate of 12.9%. Infection rates vary across regions, with 17.3% in South America, 11.5% in Asia, 10.4% in Oceania, 15.4% in Europe, 12.9% in North America, and 6.5% in Africa [43]. However, the infection rate in Yunnan was only 0.59% [44].

This study demonstrates that the infection rates of *Cryptosporidium* spp., *G. duodenalis*, and *E. bieneusi* in Guizhou are higher than those in Sichuan and Yunnan. Guizhou has a subtropical humid monsoon climate, with an annual temperature of 9 °C to 18 °C and an average annual precipitation of 665–1159 mm, with humidity ranging from 68.0% to 87.0% (The data is sourced from the China Meteorological Data Network, https://data.cma.cn/site/index.html, accessed on 8 July 2025). The average annual temperature in Sichuan is 16–18 °C in the basin, 4–12 °C in the western plateau, with an average annual precipitation of 800–1200 mm in the basin area and 400–600 mm in the western plateau, and humidity ranging from 70.0% to 80.0% in the basin and 50.0% to 60.0% in the western plateau (the data is sourced from the Sichuan Meteorological Bureau, http://sc.cma.gov.cn, accessed on 10 March 2025). Yunnan has an average annual temperature of 10 °C to 20 °C, with precipitation ranging from 800 to 2000 mm and humidity of 60.0–80.0% (The data are sourced from the Yunnan Meteorological Bureau, http://yn.cma.gov.cn, accessed on 10 March 2025). In contrast, Sichuan has lower humidity and temperature, which is less favorable for parasite prevalence. The humid environment in Guizhou provides stable conditions for the survival of *Cryptosporidium* spp. oocysts, *G. duodenalis* cysts, and *E. bieneusi* spores. In some areas of Sichuan and Yunnan, the long drought season and strong ultraviolet rays are not conducive to parasite survival. Guizhou’s karst landform also contributes to the contamination of drinking water sources [45,46,47].

### 4.2. From Animal-Adapted to Zoonotic: Evolutionary Risks of G. duodenalis Assemblage E

The zoonotic *G. duodenalis* assemblage A, detected in this study, is occasionally found in ruminants. Among all *G. duodenalis* isolates detected in Chinese cattle, assemblage A constitutes 8.5%, a relatively small proportion. Assemblage A has been detected in cattle in regions, including Shaanxi [14], Shanghai [16], Hebei [18], Guangdong [21], Inner Mongolia [28], Shanxi [30], Xinjiang [48], Henan [49], Sichuan [50], Qinghai [51], Jiangxi [52], and Yunnan [53]. Despite the low prevalence of zoonotic *G. duodenalis* assemblage A in cattle, its potential for zoonotic transmission cannot be overlooked. Among the detected *G. duodenalis* isolates, assemblage E was predominant, consistent with the prevalence pattern in cattle. Although no large-scale human infections with assemblage E have been reported, the frequent chromosomal rearrangements in *G. duodenalis* suggest the possibility of assemblage E adapting to human hosts in the future [54,55]. Therefore, strengthening epidemiological surveillance and implementing prevention and control measures are essential.

### 4.3. BEB4/I Co-Prevalence: Waterborne Transmission in Karst Hydrology

Among the positive samples of *E. bieneusi*, three genotypes were identified: I, BEB4, and J, with I and BEB4 being the dominant genotypes. This finding is similar to reports from Shanxi, where genotype I was predominant [56]. Although genotype J was also detected in this study, it was not the dominant genotype, differing from reports in Xinjiang [57], Shandong, Guangdong, and Gansu [58], where genotype J and I were the dominant genotypes. Additionally, this differs from the northwest region, where genotype BEB6 is the dominant genotype and was not detected in this study [59].

In this study, the co-dominant genotypes I and BEB4 were identified for the first time. Possible reasons include: Guizhou’s complex groundwater and humid environment favor the survival and transmission of microsporidia oocysts. Surface runoff may carry pathogens from animal or human feces into water sources, increasing co-infection risks. High humidity may extend the survival of oocysts, promoting the spread of different genotypes. Cattle frequently contact wild animals and farms in remote mountainous areas, which may make them natural hosts of *E. bieneusi*. Genotype BEB4 is common in pigs and cattle, while genotype I is often linked to human infections, suggesting a zoonotic transmission chain. In free-range systems, poor feces management (e.g., open-air stacking) worsens cross-transmission among hosts. The regional specificity of microbial genotypes means that the first detection of BEB4 and I in Guizhou may reflect unique local ecological pressures affecting pathogen evolution. The co-prevalence of BEB4 and I may complicate treatment, as different genotypes may respond differently to anti-microsporidia drugs (e.g., albendazole). Guizhou’s rural areas have weak sanitation and medical resources, and the pathogen monitoring system is incomplete, which may delay the identification and intervention of co-prevalence. Future studies should increase sample sizes, clarify the distribution of BEB4 and I in humans, livestock, and the environment, analyze the link between water pollution and genotype co-prevalence considering Guizhou’s karst topography, promote harmless feces treatment (e.g., bio-fermentation beds), reduce environmental oocyst pollution, and strengthen farm hygiene to break the zoonotic transmission chain.

### 4.4. Implications for Sustainable Disease Control and Prevention

Our findings highlight two critical leverage points for parasite control: the high infection burden in 4–8-week-old Angus calves and the co-circulation of zoonotic *E. bieneusi* assemblages. We propose a tiered intervention framework: (1) Oral administration of a formulated tebufenozide-albendazole premix significantly reduces the intestinal parasitic protozoan burden in Angus calves aged 4–8 weeks [60,61]. (2) Priority segregation of Angus calves aged 4–8 weeks, coupled with bio-fermentation to disrupt oocyst environmental persistence, temperature ≥ 55 °C for 3 days [62]. (3) Protection of karst water sources through filtration and UV disinfection, 1 mJ/cm^2^, due to the high humidity and hydrological vulnerability of Guizhou [63,64,65]. (4) Perform antibody-based screening for *Cryptosporidium* spp., *G. duodenalis*, and *E. bieneusi* among occupationally exposed groups, notably agricultural and abattoir personnel. Such integrated strategies could reduce parasite transmission while aligning with local agricultural practices.

## 5. Conclusions

This study represents the first systematic investigation of the prevalence and genotype distribution of three zoonotic parasites (*Cryptosporidium* spp., *G. duodenalis*, and *E. bieneusi*) in Angus calves in Guizhou Province, China. It was found that *Cryptosporidium* species had a 23.5% infection rate, predominantly *C. bovis* (89.6%), with significant geographical variation and higher rates in Angus calves aged 4–8 weeks (30.1%), but no association with diarrhea (*p* = 0.357). The geographical distribution characteristics of *C. bovis* subtypes (XXVIa-XXVIf) were discovered, providing a molecular basis for tracking the transmission path. Additionally, *G. duodenalis* showed a 31.3% infection rate, dominated by animal-adapted assemblage E (97.6%), suggesting that continuous monitoring of the adaptive evolution of *G. duodenalis* assemblage E is necessary to prevent future zoonotic risks. Moreover, *E. bieneusi* had a 19.7% infection rate, exclusively zoonotic genotypes (BEB4: 49.7%; type I: 40.4%), and was strongly associated with diarrhea (*p* < 0.001), highlighting its clinical significance and potential public health risks. It is recommended to strengthen prevention and control measures for Angus calves aged 4–8 weeks and optimize farm management (such as group feeding) to reduce cross-infection.

## Figures and Tables

**Table 1:** Sequence information of the primers used for PCRs in this study.

Targets	Primer Names	Sequence (5′-3′)	Annealing Temperature
18rRNA [33]	18S-F1	TTCTAGAGCTAATACATGCG	55 °C
	18S-R1	CCCATTTCCTTCGAAACAGGA	
	18S-F2	GGAAGGGTTGTATTTATTAGATAAAG	
	18S-R2	CTCATAAGGTGCTGAAGGAGTA	
*gp60* [34]	Bovis-*gp60*-F1	ATGCGACTTACGCTCTACATTACTCT	
	Bovis-*gp60*-R1	GACAAAATGAAGGCTGAGATAGATGGGA	
	Bovis-*gp60*-F2	CCTCTCGGCATTTATTGCCCT	
	Bovis-*gp60*-R2	ATACCTAAGGCCAAATGCTGATGAA	
*bg* [35]	*bg*-F1	AAGCCCGACGACCTCACCCGCAGTGC	65 °C
	*bg*-R1	GAGGCCGCCCTGGATCTTCGAGACGAC	
	*bg*-F2	GAACGAGATCGAGGTCCG	55 °C
	*bg*-R2	CTCGACGAGCTTCGTGTT	
*gdh* [35]	*gdh*-F1	TTCCGTGTCCAGTACAACTC	50 °C
	*gdh*-R1	GCCAGCTTCTCCTCGTTGAA	
	*gdh*-F2	CGCTTCCACCCCTCTGTCAAT	
	*gdh*-R2	TGTTGTCCTTGCACATCTC	
*tpi* [35]	*tpi-F1*	AATAAATIATGCCTGCTCGTCG	54 °C
	*tpi-R1*	ATGGACITCCTCTGCCTGCTC	
	*tpi*-F2	CCCTTCATCGGIGGTAACTTCAA	58 °C
	*tpi*-R2	GTGGCCACCACICCCGTGCC	
ITS [36]	Eb-ITS-F1	GATGGTCATAGGGATGAAGAGCTT	55 °C
	Eb-ITS-R1	TATGCTTAAGTCCAGGGAG	
	Eb-ITS-F2	AGGGATGAAGAGCTTCGGCTCTG	
	Eb-ITS-R2	AGTGATCCTGTATTAGGGATATT	

**Table 2:** Geographical distribution of *Cryptosporidium* spp. prevalence.

Factors	No. Tested	No. Positive	*p* Value	Species (n)
		(%, 95% CI)		
Location				
Hongling	174	21 (12.1, 7.7–17.9)	<0.001	*C. bovis* (19), *C. ryanae* (1), *C. bovis* & *C. ryanae* (1)
Wuli	168	47 (27.9, 21.3–35.3)		*C. bovis* (42), *C. ryanae* (2), *C. andersoni* (3)
Tieshi	102	34 (33.3, 24.3–43.3)		*C. bovis* (28), *C. ryanae* (6)
Guanxin	93	18 (19.4, 11.9–28.9)		*C. bovis* (18)
Gantang	100	26 (26.0, 17.7–35.7)		*C. bovis* (23), *C. ryanae* (2), *C. andersoni* (1)
Guangyindong	97	28 (28.9, 20.1–39.0)		*C. bovis* (24), *C. ryanae* (4)
Jinbi	83	18 (21.7, 13.4–32.1)		*C. bovis* (18)
Season				
spring	663	155 (23.4, 20.2–26.8)	0.963	*C. bovis* (141), *C. ryanae* (12), *C. andersoni* (1), *C. bovis* & *C. ryanae* (1)
summer	154	37 (24.0, 17.5–31.6)		*C. bovis* (31), *C. ryanae* (3), *C. andersoni* (3)
Age (week)				
0–2	107	2 (4.7, 1.5–10.6)	<0.01	*C. bovis* (2)
2–4	200	43 (21.5, 16.0–27.8)		*C. bovis* (41), *C. ryanae* (2)
4–8	479	143 (29.9, 25.8–34.1)		*C. bovis* (126), *C. ryanae* (13), *C. andersoni* (3), *C. bovis* & *C. ryanae* (1)
8–12	31	4 (12.9, 3.6–29.8)		*C. bovis* (3), *C. andersoni* (1)
Diarrhea				
Yes	93	21 (22.6, 14.6–32.4)	0.817	*C. bovis* (18), *C. andersoni* (3)
No	724	171 (23.6, 20.6–26.9)		*C. bovis* (154), *C. ryanae* (12), *C. andersoni* (4), *C. bovis* & *C. ryanae* (1)
Gender				
Male	178	32 (17.9, 12.6–24.4)	0.032	*C. bovis* (26), *C. ryanae* (4), *C. andersoni* (2)
Female	639	160 (25.0, 21.7–28.6)		*C. bovis* (146), *C. ryanae* (11), *C. andersoni* (2), *C. bovis* & *C. ryanae* (1)
Total	817	192 (23.5, 20.6–26.6)		*C. bovis* (172), *C. ryanae* (15), *C. andersoni* (4), *C. bovis* & *C. ryanae* (1)

**Table 3:** The distribution of subtypes of *Cryptosporidium bovis.*

Farm	Subtypes (n)
Hongling	XXVIf (18)\XXVIe (1)
Wuli	XXVIb (13)\XXVIc (5)\XXVId (11)\XXVIe (12)\mixed infection(XXVIb and XXVIc) (1)
Tieshi	XXVIe (23)\XXVIf (5)
Guanxin	XXVIe (13)\XXVIf (5)
Gantang	XXVIa (1)\XXVId (21)\XXVIf (2)
Guangyindong	XXVIa (12)\XXVId (10)\XXVIe (1)\XXVIf (1)
Jinbi	XXVIb (1)\XXVIc (17)

**Table 4:** Assemblage detected and positive rates of *Giardia duodenalis* in different farms in Guizhou.

Factors	No. Tested	No. Positive	*p* Value	Assemblages (n)		
		(%, 95% CI)				
Location				*bg* gene (n)	*gdh* gene (n)	*tpi* gene (n)
Hongling	174	33 (18.9, 13.4–25.6)	<0.001	A (2), E (22)	E (18)	A (2), E (15)
Wuli	168	44 (26.2, 19.7–33.5)		A (2), E (34)	A (3), E (23)	A (1), E (18)
Tieshi	102	5 (4.9, 1.6–11.1)		E (5)	E (4)	E (3)
Guanxin	93	37 (39.8, 29.8–50.5)		A (1), E (30)	A (1), E (23)	E (21)
Gantang	100	55 (55.0, 44.7–65.0)		E (42)	E (34)	E (18)
Guangyindong	97	50 (51.6, 41.2–61.8)		E (37)	E (26)	E (16)
Jinbi	83	32 (38.5, 28.1–49.9)		E (23)	E (12)	E (11)
Season						
spring	663	224 (33.8, 30.2–37.5)	0.569	E (219), A (5)		
summer	154	32 (20.8, 14.7–28.0)		E (31), A (1)		
Age (week)				Assemblage		
0–2	107	19 (17.7, 11.0–26.3)	<0.001	E (19)		
2–4	200	49 (24.5, 18.7–31.1)		E (48), A (1)		
4–8	479	178 (37.2, 32.8–41.7)		E (173), A (5)		
8–12	31	10 (32.2, 16.7–51.4)		E (10)		
Diarrhea				Assemblage		
Yes	93	23 (24.7, 16.3–34.8)	0.166	E (23)		
No	724	233 (32.2, 28.8–35.7)		E (227), A (6)		
Gender				Assemblage		
Male	178	53 (29.8, 23.2–37.1)	0.629	E (52), A (1)		
Female	639	203 (31.8, 28.2–35.5)		E (198), A (5)		
Total	817	256 (31.3, 28.1–34.6)		E (250), A (6)		

**Table 5:** Genotypes and positive rates of *Enterocytozoon bieneusi* in different farms in Guizhou.

Factors	No. Tested	No. Positive	*p* Value	Genotypes (n)
		(%, 95% CI)		
Location				
Hongling	174	0 (0.0, 0.0–2.1)	<0.001	-
Wuli	168	31 (18.5, 12.9–25.2)		I (11), BEB4 (7), J (13)
Tieshi	102	24 (23.5, 15.7–32.9)		BEB4 (24)
Guanxin	93	0 (0.0, 0.0–3.9)		-
Gantang	100	39 (39.0, 29.4–49.3)		I (27), BEB4 (9), CHPM1 (3)
Guangyindong	97	38 (39.2, 29.4–49.6)		I (13), BEB4 (25)
Jinbi	83	29 (34.9, 24.8–46.2)		I (14), BEB4 (15)
Season				
spring	663	142 (21.4, 18.4–24.7)	0.843	I (59), BEB4 (80), CHPM1 (3)
summer	154	19 (12.0, 7.6–18.6)		I (6), J (13)
Age (week)				
0–2	107	7 (6.5, 2.7–13.0)	<0.001	I (5), BEB4 (2)
2–4	200	25 (12.5, 8.3–17.9)		I (11), BEB4 (12), CHPM1 (1), J (1)
4–8	479	125 (26.1, 22.2–30.3)		I (48), BEB4 (64), CHPM1 (2), J (11)
8–12	31	4 (12.9, 3.6–29.8)		I (1), BEB4 (2), J (1)
Diarrhea				
Yes	93	33 (35.5, 25.8–46.1)	<0.01	I (13), BEB4 (20)
No	724	128 (17.7, 15.0–20.7)		I (52), BEB4 (60), CHPM1 (3), J (13)
Gender				
Male	178	22 (12.4, 7.9–18.1)	0.065	I (8), BEB4 (11), J (3)
Female	639	139 (21.8, 18.6–25.2)		I (57), BEB4 (69), CHPM1 (3), J (10)
Total	817	161 (19.7, 17.0–22.6)		I (65), BEB4 (80), CHPM1 (3), J (13)

## Data Availability

Sequence data from this study have been submitted to NCBI (*Cryptosporidium* spp. SSU rRNA: PV740224-PV740233; *gp60*: PV764543-PV764551. *Enterocytozoon bieneusi* ITS： PV747187-PV747192; *Giardia duodenalis bg:* PV763447-PV763459; *tpi:* PV763460-PV763471; *gdh:* PV764534-PV764542. The detailed information of the sequence was described in Supplementary Table S2.

## Supplementary Materials

The following supporting information can be downloaded at: https://www.mdpi.com/article/10.3390/microorganisms13081735/s1, Figure S1. Phylogenetic analysis of representative sequences for the 18S rRNA locus of *Cryptosporidium species* in this study with referenced sequences, obtained via Neighbor—Joining analysis; Figure S2. Phylogenetic analysis of representative sequences for the gp60 locus of *Cryptosporidium bovis* in this study with referenced sequences, obtained via Neighbor—Joining analysis; Figure S3. Phylogenetic analysis of representative sequences for the bg locus of *Giardia duodenalis* in this study with referenced sequences, obtained via Neighbor—Joining analysis; Figure S4. Phylogenetic analysis of representative sequences for the tpi locus of *Giardia duodenalis* in this study with referenced sequences, obtained via Neighbor—Joining analysis; Figure S5. Phylogenetic analysis of representative sequences for the *gdh* locus of *Giardia duodenalis* in this study with referenced sequences, obtained via Neighbor—Joining analysis; Figure S6. Phylogenetic analysis of representative sequences for the ITS locus of *Enterocytozoon bieneusi* in this study with referenced sequences, obtained via Neighbor—Joining analysis; Table S1. Detailed sampling data; Table S2. Homology analysis of SSU rRNA, *gp60*, ITS, *bg*, *tpi* and *gdh* sequences of *Cryptosporidium* spp., *Enterocytozoon bieneusi* and *Giardia duodenalis*.
