# Supplementary material for: Molecular Epidemiology of Cryptosporidium spp., Giardia duodenalis, and Enterocytozoon bieneusi in Guizhou Angus Calves: Dominance of Angus Cattle-Adapted Genotypes and Zoonotic Potential of E. bieneusi
Source: Microorganisms. 2025 Jul 25;13(8):1735. doi: 10.3390/microorganisms13081735 (PMC12388192; doi:10.3390/microorganisms13081735)
Supplement: Supplementary file 1 [file microorganisms-13-01735-s001.zip › microorganisms-3726115 Supplementary Figure.pdf]

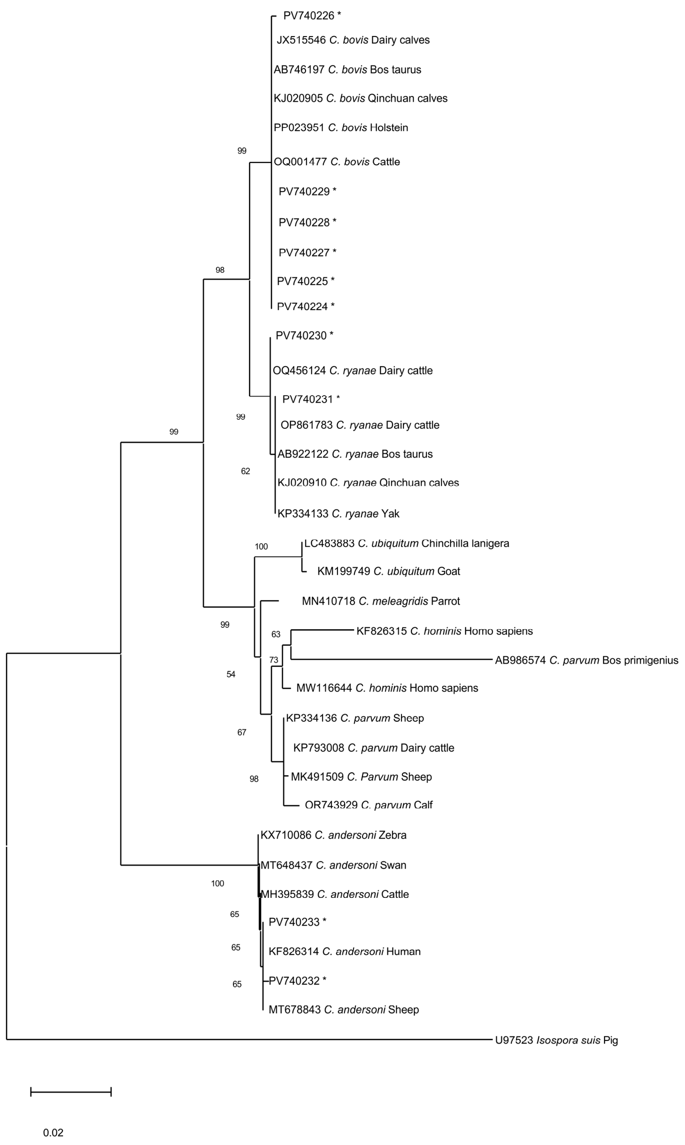

Figure S1. Phylogenetic analysis of representative sequences for the *18S* r*RNA* locus of *Cryptosporidium species* in this study with referenced sequences, obtained via Neighbor - Joining analysis. Bootstrap support values based on 1000 replicates are shown at the nodes. The sequences obtained in this study are marked with asterisk (*), *Isospora suis* (U97523) was used as the outgroup.

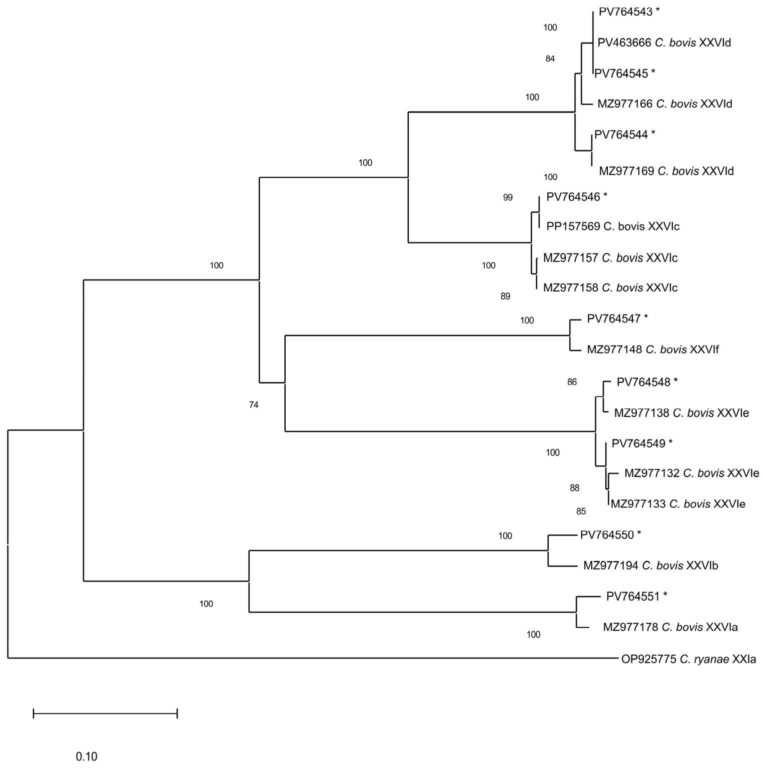

Figure S2. Phylogenetic analysis of representative sequences for the *gp60* locus of *Cryptosporidium bovis* in this study with referenced sequences, obtained via Neighbor - Joining analysis. Bootstrap support values based on 1000 replicates are shown at the nodes. The sequences obtained in this study are marked with asterisk (*). *C. ryanae* XXIa subtype (OP925775) was used as the outgroup.

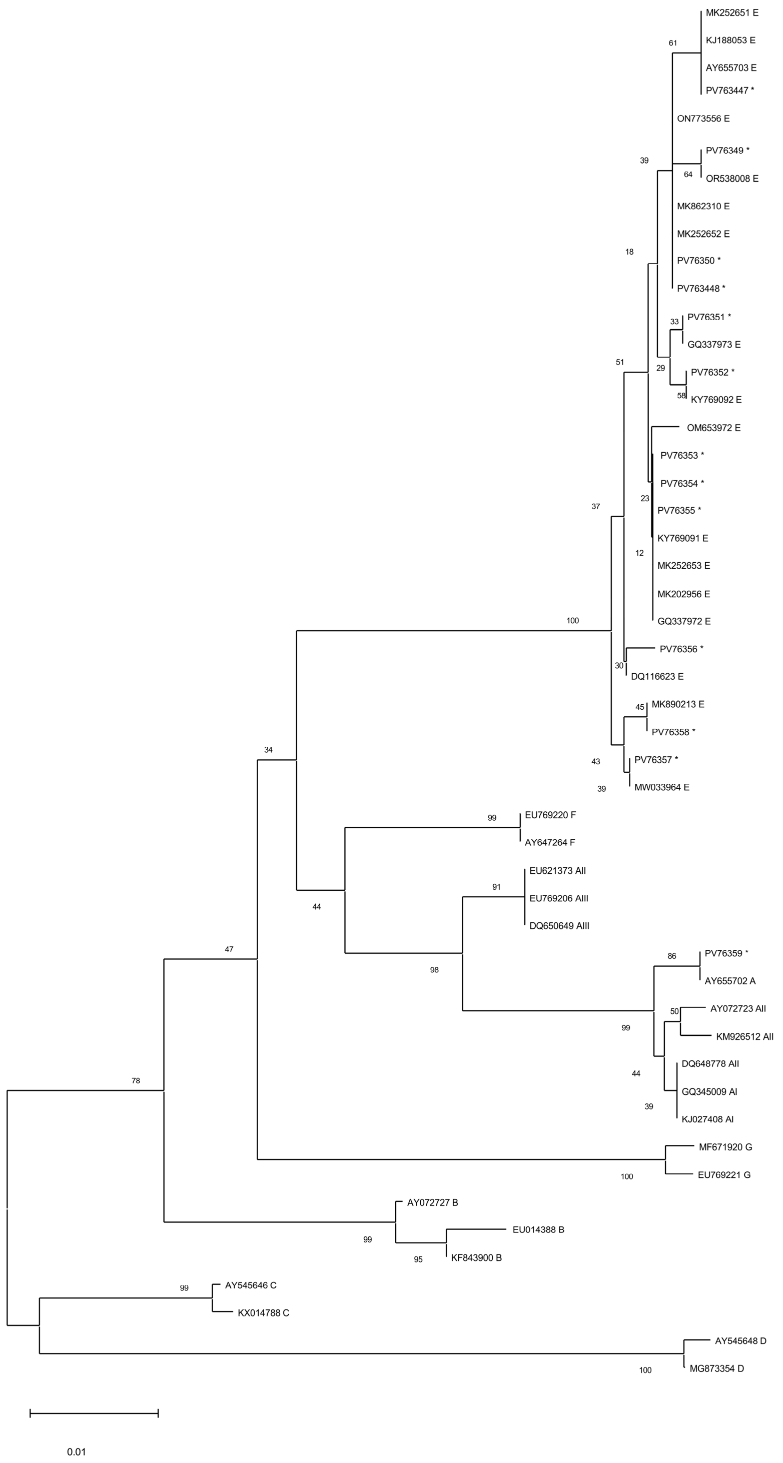

Figure S3. Phylogenetic analysis of representative sequences for the *bg* locus of *Giardia duodenalis* in this study with referenced sequences, obtained via Neighbor - Joining analysis. Bootstrap support values based on 1000 replicates are shown at the nodes. The sequences obtained in this study are marked with asterisk (*).

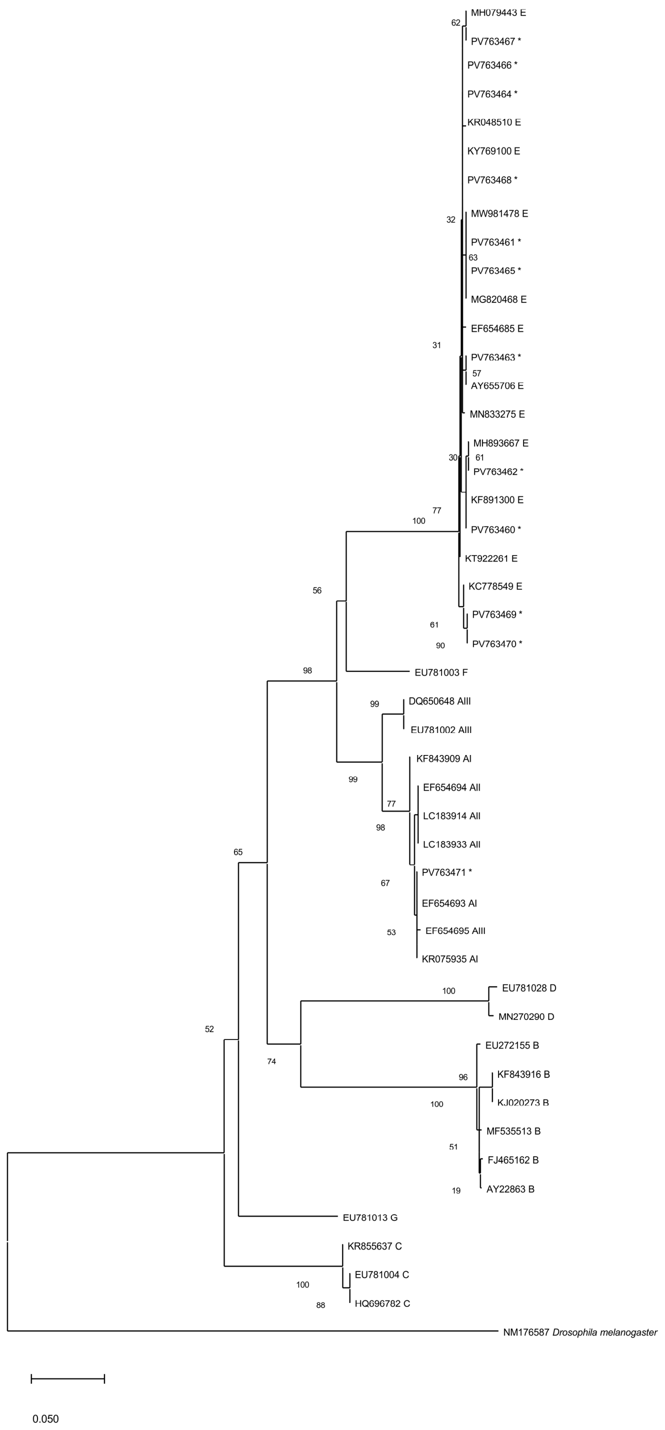

Figure S4. Phylogenetic analysis of representative sequences for the *tpi* locus of *Giardia duodenalis* in this study with referenced sequences, obtained via Neighbor - Joining analysis. Bootstrap support values based on 1000 replicates are shown at the nodes. The sequences obtained in this study are marked with asterisk (*). *Drosophila melanogaster* (NM176587) was used as the outgroup.

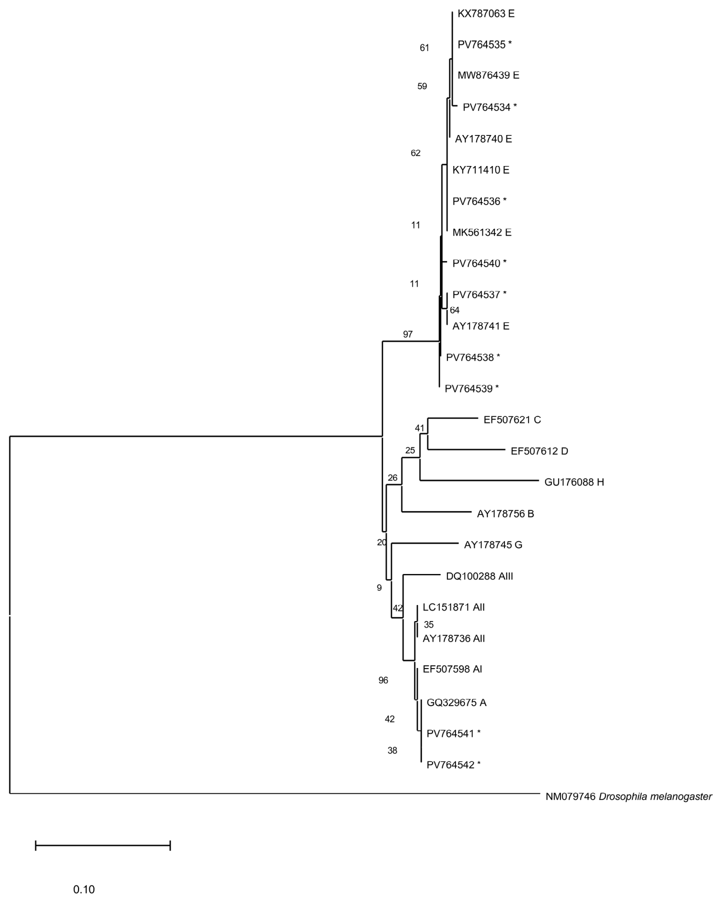

Figure S5. Phylogenetic analysis of representative sequences for the *gdh* locus of *Giardia duodenalis* in this study with referenced sequences, obtained via Neighbor - Joining analysis. Bootstrap support values based on 1000 replicates are shown at the nodes. The sequences obtained in this study are marked with asterisk (*). *Drosophila melanogaster* (NM079746) was used as the outgroup.

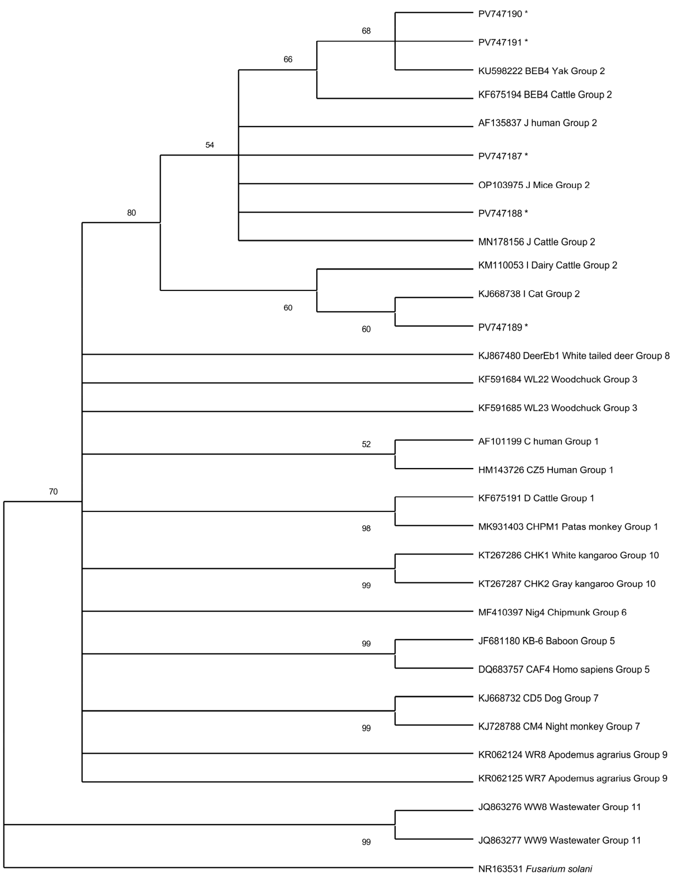

Figure S6. Phylogenetic analysis of representative sequences for the *ITS* locus of *Enterocytozoon bieneusi* in this study with referenced sequences, obtained via Neighbor - Joining analysis. Bootstrap support values (>50%) are shown at the nodes. The sequences obtained in this study are marked with asterisk (*). *Fusarium solani* (NR163531) was used as the outgroup.
