# Supplementary material for: Molecular Epidemiology of Cryptosporidium spp., Giardia duodenalis, and Enterocytozoon bieneusi in Guizhou Angus Calves: Dominance of Angus Cattle-Adapted Genotypes and Zoonotic Potential of E. bieneusi
Source: Microorganisms. 2025 Jul 25;13(8):1735. doi: 10.3390/microorganisms13081735 (PMC12388192; doi:10.3390/microorganisms13081735)
Supplement: Supplementary file 1 [file microorganisms-13-01735-s001.zip › microorganisms-3726115 Supplementary table2.pdf]

Table S2. Homology analysis of SSU rRNA, *gp60*, ITS, *bg*, *tpi* and *gdh* sequences of *Cryptosporidium* spp., *Enterocytozoon bieneusi* and *Giardia duodenalis.*

Organism	Target gene	Notes	Accession nos.[1]	Accession nos.[2]	Homology
Cryptosporidium spp.	SSU rRNA	*C. bovis*	PV740224	OQ001460	100.00%
			PV740225	OQ001477	100.00%
			PV740226	OR460664	100.00%
			PV740227	PP023998	100.00%
			PV740228	OP861737	100.00%
			PV740229	KJ020905	100.00%
		C. ryanae	PV740230	OP861797	100.00%
			PV740231	KJ020906	100.00%
		C. andersoni	PV740232	OR460762	100.00%
			PV740233	KF271479	100.00%
	gp60	XXVId	PV764543	PV463666	100.00%
			PV764544	MZ977169	98.78%
			PV764545	PV463666	100.00%
		XXVIc	PV764546	MZ977150	100.00%
		XXVIf	PV764547	MZ977149	100.00%
		XXVIe	PV764548	PP157539	100.00%
			PV764549	MZ977133	100.00%
		XXVIb	PV764550	OM136968	100.00%
		XXVIa	PV764551	MZ977188	100.00%
G. duodenalis	*bg*	Assemblage E	PV763447	AY653159	100.00%
			PV763448	DQ116615	100.00%
			PV763449	OR538008	100.00%
			PV763450	MK862310	100.00%
			PV763451	OM653973	100.00%
			PV763452	MK862312	100.00%
			PV763453	MK202956	100.00%
			PV763454	GQ337972	100.00%
			PV763455	MN833266	100.00%
			PV763456	MK642904	100.00%
			PV763457	MW033964	100.00%
			PV763458	MK890213	100.00%
		Assemblage A	PV763459	HQ538712	100.00%
	tpi	Assemblage E	PV763460	KF891300	100.00%
			PV763461	MG820468	100.00%
			PV763462	MH893667	100.00%
			PV763463	MH079445	100.00%
			PV763464	AB569406	100.00%
			PV763465	KY710747	100.00%
			PV763466	KF843945	100.00%

			PV763467	MH079443	100.00%
			PV763468	KJ917622	100.00%
			PV763469	EU189342	100.00%
			PV763470	OL456207	100.00%
		Assemblage A	PV763471	JX845433	100.00%
			PV764534	JF957622	99.71%
			PV764535	KU382251	100.00%
			PV764536	KY711410	100.00%
		Assemblage E	PV764537	AY178741	100.00%
	gdh		PV764538	JF792402	99.71%
			PV764539	MW876460	99.72%
			PV764540	GQ337965	100.00%
		Assemblage A	PV764541	GQ329675	100.00%
			PV764542	JF957621	100.00%
		J	PV747187	DQ885586	100.00%
			PV747188	PP956199	98.90%
E. bieneusi	ITS	I	PV747189	KX641288	98.29%
		BEB4	PV747190	MH732750	99.71%
			PV747191	KU598222	100.00%
		CHPM1	PV747192	MK931403	100.00%

[1]The sequences were obtained in this study.

[2]The sequence with the highest homology was selected.
